# Corrigendum

**DOI:** 10.1590/2237-6089-2022-0003

**Published:** 2023-01-04

The authors of the article entitled “Translation and cross-cultural adaptation of the Arizona Sexual Scale (ASEX) into Portuguese” (doi: http://dx.doi.org/10.1590/2237-6089-2018-0056), published in Trends in Psychiatry and Psychotherapy in volume 41 (2019), issue 3, pages 247-253, would like to correct an error in the Appendix, namely, on page 253, question no. 3 of the female version of the scale is shown in English, when instead it should be shown in Portuguese. Below we reproduce the correct version of this part of the scale.

**ESCALA DE EXPERIÊNCIA SEXUAL DE ARIZONA**

**(ASEX)** ^**©**^ **- FEMININO**

Para cada questão, por favor, indique o seu nível GLOBAL durante a SEMANA PASSADA, incluindo o dia de HOJE.

**1. Qual é a intensidade do seu impulso sexual?**

**Table:** 

1 □	2 □	3 □	4 □	5 □	6 □
extremamente forte	muito forte	relativamente forte	relativamente fraco	muito fraco	sem impulso sexual

**2. Com que facilidade se excita sexualmente?**

**Table:** 

1 □	2 □	3 □	4 □	5 □	6 □
extremamente fácil	muito fácil	relativamente fácil	relativamente difícil	muito difícil	nunca me excito

**3. Com que facilidade a sua vagina lubrifica durante o acto sexual?**

**Table:** 

1 □	2 □	3 □	4 □	5 □	6 □
extremamente fácil	muito fácil	relativamente fácil	relativamente difícil	muito difícil	nunca

**4. Com que facilidade atinge o orgasmo?**

**Table:** 

1 □	2 □	3 □	4 □	5 □	6 □
extremamente fácil	muito fácil	relativamente fácil	relativamente difícil	muito difícil	nunca me excito

**5. Os seus orgasmos são compensadores?**

**Table:** 

1 □	2 □	3 □	4 □	5 □	6 □
bastante compensadores	muito compensadores	relativamente compensadores	algo insatisfatórios	muito insatisfatórios	não consigo alcançar atingir o orgasmo

**COMENTÁRIOS:**

