# Corrigendum

**DOI:** 10.47626/2237-6089-2023-0002

**Published:** 2023-11-13

We used the wrong DOI prefix in the Corrigendum e20220003, published in volume 45.

Wrong/old DOI number: https://doi.org/10.1590/2237-6089-2022-0003

Correct DOI number: https://doi.org/10.47626/2237-6089-2022-0003

